# Estimation of Number of Graphene Layers Using Different Methods: A Focused Review

**DOI:** 10.3390/ma14164590

**Published:** 2021-08-16

**Authors:** Vineet Kumar, Anuj Kumar, Dong-Joo Lee, Sang-Shin Park

**Affiliations:** 1 School of Mechanical Engineering, Yeungnam University, Gyeongsan 38541, Korea; vineetfri@gmail.com (V.K.); djlee@yu.ac.kr (D.-J.L.); 2 School of Chemical Engineering, Yeungnam University, Gyeongsan 38541, Korea; anuj.budhera@gmail.com

**Keywords:** graphene, properties, sp^2^ hybridized carbon atoms, synthesis, applications

## Abstract

Graphene, a two-dimensional nanosheet, is composed of carbon species (sp^2^ hybridized carbon atoms) and is the center of attention for researchers due to its extraordinary physicochemical (e.g., optical transparency, electrical, thermal conductivity, and mechanical) properties. Graphene can be synthesized using top-down or bottom-up approaches and is used in the electronics and medical (e.g., drug delivery, tissue engineering, biosensors) fields as well as in photovoltaic systems. However, the mass production of graphene and the means of transferring monolayer graphene for commercial purposes are still under investigation. When graphene layers are stacked as flakes, they have substantial impacts on the properties of graphene-based materials, and the layering of graphene obtained using different approaches varies. The determination of number of graphene layers is very important since the properties exhibited by monolayer graphene decrease as the number of graphene layer per flake increases to 5 as few-layer graphene, 10 as multilayer graphene, and more than 10 layers, when it behaves like bulk graphite. Thus, this review summarizes graphene developments and production. In addition, the efficacies of determining the number of graphene layers using various characterization methods (e.g., transmission electron microscopy (TEM), atomic force microscopy (AFM), scanning electron microscopy (SEM), X-ray diffraction (XRD), Raman spectra and mapping, and spin hall effect-based methods) are compared. Among these methods, TEM and Raman spectra were found to be most promising to determine number of graphene layers and their stacking order.

## 1. Introduction

The 6th element of the periodic table is truly fascinating [1]. From the perspective of graphene chemistry, carbon has several allotropes and, when combined with various organic and inorganic molecules, can form an almost infinite number of hybrids. For example, it can be used to produce a soft lubricating material, such as graphite, and at the other end of the scale it can form diamond, the hardest material known. The most famous allotropes of carbon are 0-dimensional (0D) fullerenes [2], 1D carbon nanotubes [3], and 2D graphene [4]. Graphene is perhaps the most interesting carbon allotrope from the viewpoint of research and potential applications due to its fascinating properties [4,5]. Graphene can be categorized based on the number of graphene layers (L), stacked as monolayer graphene (1 L), few-layer graphene (<5 L), multilayer graphene (<10 L), or graphene nanoplatelets (>10 L) [6].

Around 70 years ago, scientists debated whether two-dimensional (2D) materials existed due to their thermodynamical instability [7,8]. Graphene (or “2D graphite”) was considered for 60 years [9,10,11] and was used to describe the properties of various carbon-based materials. Forty years later, it was understood that graphene provides a good condensed-matter analog of (2 + 1)-dimensional quantum electrodynamics [12,13,14], and, in 2004, it was synthesized by exfoliation of HOPG using adhesive tape. This innovation has since fueled a great number of investigations aimed at understanding the unique electronic structure of graphene. Ganguly et al. described that colloidal graphene is a key component for the molecular dispersion of graphene sheets [15].

Graphite has a 3D structure with large particle sizes and a small surface area, whereas graphene is monoatomic in thickness and has small particle sizes and an extremely high surface area; these characteristics have remarkable effects on its properties [4,5]. Accordingly, extensive research has been conducted to produce graphene in bulk for various applications, especially for electronics and batteries. Several studies have been performed to produce graphene flakes with different numbers of layers. Today graphene can be synthesized using top-down [5,16,17,18,19,20,21] or bottom-up approaches [15,22,23,24,25,26]. Top-down approaches include scotch tape exfoliation [5], liquid-phase exfoliation in different solvents [18], or chemical synthesis using redox reactions [19,20,21], whereas bottom-up approaches include chemical vapor deposition (CVD) [22,23,24] and molecular beam epitaxy [25,26].

Regardless of the method used, the number of graphene layers per flake, importantly, determines the properties of the produced materials. Before we describe earlier work on graphene, one needs to understand what a 2D crystal is. Atoms arranged regularly in a single plane form a 2D crystal, and 100 layers of such planes would be considered a thin 3D film, which prompts the question of “how many layers are needed to make a 3D structure?” It has been shown that electronic structures rapidly evolve with the number of layers and graphite approaches the 3D limit at 10 layers [27]. In this review, we focus on the techniques that are commonly used to determine the number of graphene layers. Obraztsova et al. provided a statistical analysis of AFM and Raman spectra for the determination of the number of graphene layers in graphene flake [28]. Ganguly et al. further characterized nanoparticles decorated with reduced graphene oxide and TEM investigations showed that the distinct nanoparticles were in range of 12–35 nm over reduced graphene oxide [29]. Plachá et al. reviewed graphene-based materials for various applications, such as biomedical applications [30]. Kamedulski et al. described an effective method for obtaining N-doped graphene using gamma radiation [31]. Yu et al. demonstrate the role of oxygen containing functional groups in performing water purification for graphene-oxide-based membranes [32].

## 2. Production of Graphene

**Figure 1:**
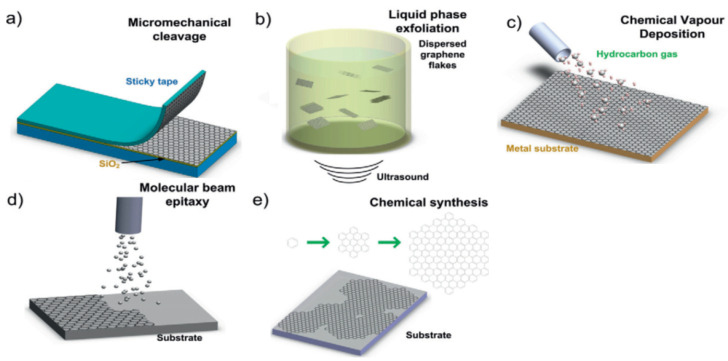
Graphene production methods (reproduced with permission from ref. [33], Copyright 2012 Elsevier).

Monolayer graphene can be produced in various ways, but is usually produced by micromechanical cleavage or liquid phase exfoliation (top-down methods), or using chemical vapor deposition (bottom-up method):

(a) Micromechanical cleavage (MMC): MMC has been used for decades to synthesize graphene (Figure 1). Geim et al. demonstrated that scotch tape could be used to micromechanically exfoliate graphite to produce graphene [5] and, in 2004, Novoselov et al. reported the isolation of monolayer graphene by mechanical exfoliation HOPG using adhesive tape [5]. MMC is now a standard method for producing defect-free monolayer graphene at a small-scale [17]. The extent of exfoliation achieved can be determined using different techniques, such as AFM or Raman spectra, as described in Section 4. Although this method is unsuitable for large-scale synthesis of graphene, it is widely used in small-scale studies.

(b) Liquid-phase exfoliation (LPE): High-quality graphene can be obtained by exfoliating bulk graphite by mechanical shear in a liquid (Figure 1). LPE of graphite into graphene has been used to produce graphene for many years. The technique was pioneered by Coleman et al. who demonstrated that sonication of graphite in a solvent produces dispersed graphene flakes [17]. LPE relies on energetically favorable interactions between a solvent and graphene sheets [34], and, thus, solvent–graphene interactions must be equivalent to the exfoliation energy of graphene [35,36]. LPE is performed in three steps: (a) dispersion of graphite in solvent; (b) sonication of the suspended graphite to cause exfoliation; and (c) centrifugation and further purification of the produced suspended graphene flakes. A number of dispersing solvents are used in this process and N-methyl pyrrolidone (NMP) and dimethyl formamide (DMF) are among the most commonly used [37]. Ultrasonication creates mechanical waves in a liquid medium, and cavitation during rarefaction cycles creates negative acoustic pressures and transient microbubbles (cavities) that separate graphene layers. Because graphene is hydrophobic, it cannot be dispersed in water without additives. These additives facilitate exfoliation and influence the restacking process. However, they must be subsequently removed by processes such as annealing. LPE provides a simple means of producing single graphene layers but yields are far too low for industrial-scale operations. The various techniques used to characterize exfoliated graphenes obtained by LPE are detailed in Section 4.

(c) Chemical vapor deposition (CVD): CVD involves the deposition of thin films from solid, liquid, or gaseous precursors. Upon heating, carbon-based compounds decompose and the carbon diffuses through and saturates metal lattices. Upon cooling, the carbon is expressed from the metal surface and forms graphene. This process can be used to manufacture large quantities of graphene (Figure 1). Several research groups are currently working on the use of CVD to synthesize single-layer graphene and have demonstrated that CVD offers a promising route for the production of defect-free graphene [22,23,24]. Several CVD techniques, including thermal and plasma-enhanced techniques, can be used to make graphene [23,24]. Process costs are an essential aspect of CVD processes, and plasma-enhanced CVD is the most cost-effective process to date [24].

(d) Molecular beam epitaxy (MBE): MBE is used widely for growing graphene layers of high carbon purity (Figure 1) on many substrates at high temperatures [25]. At lower temperatures, defects are produced in graphene synthesized using MBE [26]. MBE can be used to produce single-layer graphene of equivalent quality to MMC and the other methods discussed above.

(e) Chemical synthesis of graphene: Chemical methods have been widely used to synthesize graphene in high yields and throughputs (Figure 1). Exfoliation can be promoted by chemically transforming graphite. The techniques usually used involve chemical oxidation and exfoliation of graphite using Hummer’s method [38]. Chemical graphene synthesis is usually used to produce graphene in bulk, which involves the oxidation of graphite and its subsequent reduction using ascorbic acid [19], hydrazine [20], or other reducing agents. The oxidation of graphite can be achieved using different methods, which have been compared in a previous study [21]. Chemical synthesis provides a promising means of producing high purity, low-defect graphene at industrial levels.

## 3. Significance of the Number of Graphene Layers

### 3.1. Properties vs. Numbers of Graphene Layers

Monolayer graphene has exceptional mechanical, electrical, and thermal properties [5] and it has been well established that these properties decrease as number of graphene layers increases. Thus, it is important that the number of graphene layers be optimized for different applications.

#### 3.1.1. Mechanical Properties and Number of Layers

Mechanical properties are well known to be strongly influenced by graphene layer numbers. For example, Zhang et al. studied the effects of the number of layers on mechanical properties [39]. The results obtained showed that tensile strength and elongation at break decreased with increasing graphene layer numbers of graphene flakes. Multilayer and monolayer graphene differ in terms of stacking order; however, this interaction between adjacent layers is not strong, and thus, it does not markedly increase fracture stress or strain. Young’s modulus, however, increases with the increasing layer number in graphene flake, which means that interlayer interactions have a positive effect on the Young’s modulus of multilayer graphene [39].

**Figure 2:**
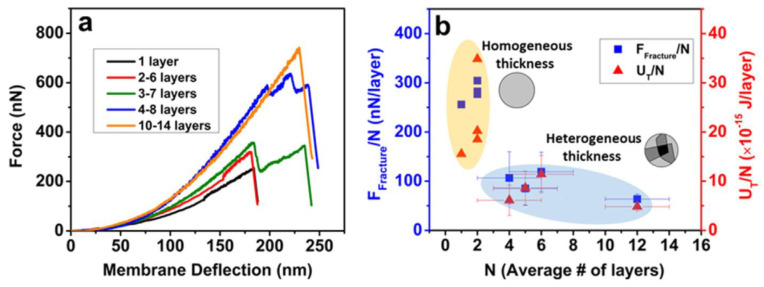
Effects of layer number on mechanical properties: (**a**) force against membrane deflection for different graphene flakes, and (**b**) mechanical properties for different graphene layer numbers (reproduced with permission from ref. [41], Copyright 2018, Elsevier).

Figure 2a shows force versus deflection plots for a graphene oxide (GO) monolayer and for four other few or multi-layered GO flakes, as determined by AFM [40]. The plots show that membrane deflection increases with the number of graphene layers. Similarly, Figure 2b shows that fracture forces were higher for few and multilayer, homogenously thick graphene layers than for graphene layers with variable thicknesses [40]. Homogenous and mono-bilayer graphene flake had the highest fracture force of all samples studied and this gradually reduced with layer number [39,40].

#### 3.1.2. Effects of Layer Number on Thermal Properties

**Figure 3:**
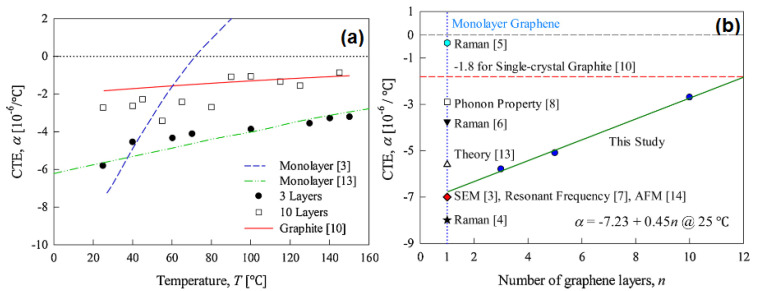
Thermal properties: (**a**) CTE as a function of temperature and (**b**) CTE as a function of layer number (reproduced with permission from ref. [42], Copyright 2020, Elsevier).

Coefficients of thermal expansion (CTE) were found to depend on temperature and layer number. Figure 3a shows the effects of temperature and the number of graphene layers on CTE. Figure 3a shows that, as temperature increases from 20 to 140 °C, the CTE increases linearly. Furthermore, monolayer graphene had the highest CTE vs. temperature slope [42]. The CTE vs. temperature plot of 10-layer graphene showed greater scatter than the less layered graphenes. However, the temperature dependencies of 10-layer and less layered graphenes were similar and CTEs were studied at 25 °C and as a function of graphene layer number, (Figure 3b) it was found that they increased from monolayer graphene up to 10 layers [42].

#### 3.1.3. Optical Properties and Number of Layers

**Figure 4:**
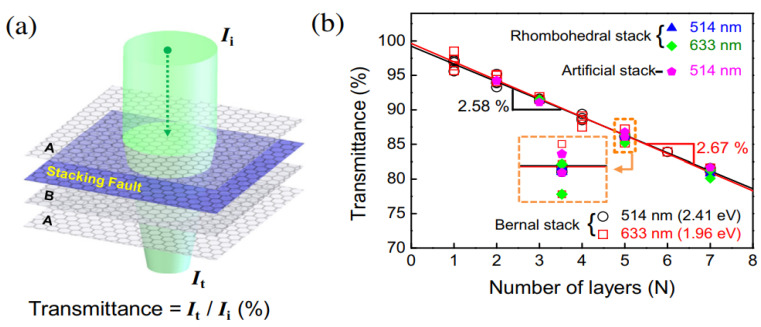
Optical transmittance measurements: (**a**) optical transmittance of multilayer graphene and (**b**) transmittance measurements for different layer numbers according to stacking order (reproduced with permission from ref. [40], Copyright 2014, Elsevier).

Optical transmittance was found to depend on layer number (Figure 4). A graphene membrane’s optical transmittance is defined as the ratio of transferred to inputted laser strength (Figure 4) [40]. It was found that optical transmission decreases with increasing layer numbers, from 1 to 7, regardless of the laser wavelength [40].

### 3.2. Restacking and Intercalation for Different Layer Numbers

Restacking involves the auto assembly of graphene layers and reduces the properties of graphene in various industrial applications, such as barrier properties in batteries. One approach involves increasing the interlayer spacing using intercalants and then removing them by rapid evaporation. For example, when graphite is soaked in a strong acid (the intercalating agent), the acid penetrates the layers and increases the interlayer spacing. Promptly increasing the temperature can cause intercalant removal, and leave behind exfoliated sheets of graphene [43]. Furthermore, the yields of these methods can be increased by ultrasonication [44].

## 4. Techniques Used to Determine Numbers of Graphene Layers

### 4.1. Transmission Electron Microscopy

**Figure 5:**
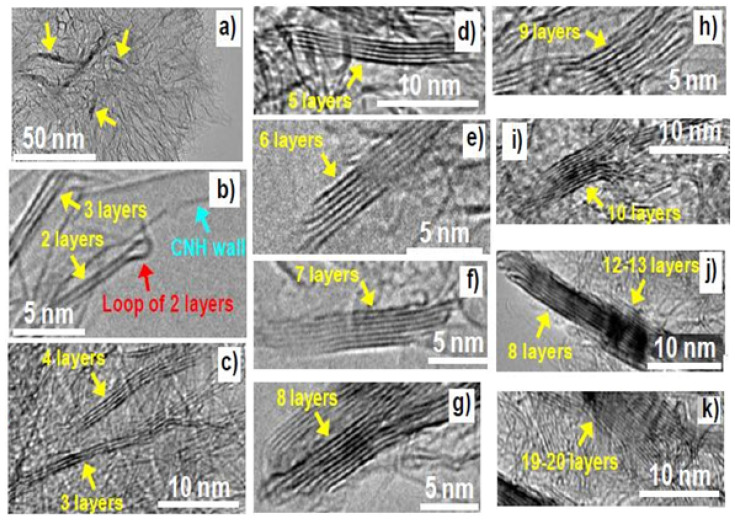
TEM and the determination of numbers of graphene layers per flake: (**a**–**k**) the images show flakes comprised of 1 to 19–20 layers (reproduced with permission from ref. [45], Copyright 2013, Elsevier).

TEM involves the transmission of a beam of electrons through an ultra-thin (<100 nm thick) specimen. TEM can provide high-resolution images down to the atomic level, and is frequently used to determine the number of graphene layers per flake. Nakamura et al. used TEM to study evenly stacked graphite-like thin sheets (GLSs) prepared by the laser ablation of graphite in argon [45]. The produced GLSs were neat, untouched, and free-standing. The authors discovered that even layer numbers (2, 4, 6, and 8) were preferentially formed [45]. Figure 5 shows the use of TEM to determine graphene layer numbers [45]. Another interesting observation was that interlayer distances decreased from 0.385 to 0.335 nm with increasing layer numbers [45]. Navik et al. studied the ultrasonication-induced exfoliation of graphite to graphene in solvent containing curcumin via TEM [46]. They found that this process produced large areas of thin, transparent graphene sheets, 1–5 graphene layers thick [46]. Cui et al. studied the effects of stacking and lattice orientation on the mechanical properties of few-layer GO [40]. They found graphene flakes were 1 to 5 layers thick and they studied the effects of the graphene layer number on the mechanical properties and found that the mechanical properties were affected by increasing layer numbers, from 1 to 5 [40]. Stobinski et al. studied graphene oxide and reduced graphene oxide using XRD, TEM, and other electron spectroscopic methods [47] and found that a reduction of graphene oxide reduced graphene interlayer spacing [47].

Al-Hagri used CVD to synthesize monolayer graphene, which was then studied using TEM [48]. The produced vertically aligned graphene nanosheets exhibited high surface areas, good mechanical strengths, excellent electric properties, and high chemical stability, which made them useful candidates for various industrial applications, such as supercapacitors and batteries [48]. Ding et al. produced high sulfonic acid edge functionalized graphene from graphite in one step [49] and used HR-TEM to determine layer numbers. The authors found mono bilayer graphene might be useful for various applications, including capacitator fabrication [49]. In addition, electron diffraction pattern spot intensities were used to confirm HR-TEM determined numbers of functionalized graphene layers [49].

### 4.2. Scanning Electron Microscopy (SEM)

**Figure 6:**
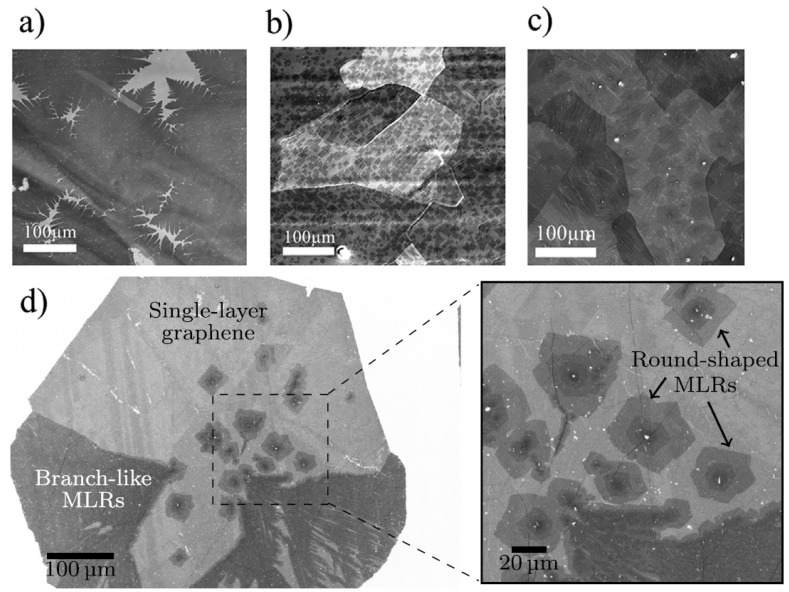
SEM micrographs of graphenes with different layer numbers: (**a**) single-layer graphene, (**b**) bi-layer graphene, (**c**) tri-layer graphene [50], (**d**) large single layer graphene sheet and multi-layer regions (MLRs) (reproduced with permission from ref. [51], Copyright 2018, Elsevier).

Scanning electron microscopes provide images by scanning sample surfaces with a focused beam of electrons. These electrons interact with atoms and produce signals that contain information about surface morphologies. SEM is frequently used to determine the number of layers in graphene flakes. Ying et al. synthesized bi-layer and multi-layer graphenes on Cu foil in one step [50]. Figure 6 shows SEM images of mono- (Figure 6a,d), bi- (Figure 6b), and tri-layered graphenes (Figure 6c) [50]. Huet et al. used CVD to synthesize graphene flakes with few defects and controllable numbers of layers and thicknesses, and demonstrated the relationship between thermal conditions and graphene growth [51]. In addition, they used SEM to characterize single-layer graphene, MLRs (multi-layer regions), and branch-like MLRs [51]. Lin et al. also used SEM to estimate the number of graphene layers [52] and reported graphene layers stacked 6 to 20 layers thick, and that ball milling for 8 h instead of 2 reduced the layer numbers from 20 to 6 [52]. Yoshihara et al. produced SLG (single-layer graphene) by chemically etching copper foils grown using CVD and characterized them by SEM, and showed that etching conditions strongly influenced graphene domain size [53]. Mohanty et al. studied FLG (few-layer graphene), prepared at two different temperatures, using SEM microscopy [54]. The benefits of a high surface-to-volume ratio 2D NP geometry are supported by mathematical calculations [54]. In another study, SLG was grown on Ge (100) by CVD and characterized by SEM [55]. Measurements showed that large areas of good quality, homogeneous monolayer graphene can be directly produced on Ge substrates.

### 4.3. Atomic Force Microscopy

**Figure 7:**
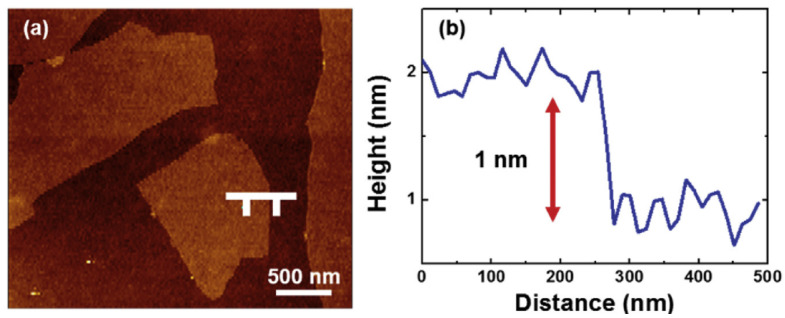
AFM images used to determine numbers of graphene layers: (**a**) topological features of single-layer graphene; and (**b**) histogram showing evidence of monolayer graphene (reproduced with permission from ref. [56], Copyright 2017, Elsevier).

AFM is a high-resolution type of microscopy with a resolution at the atomic level. AFM can be used to produce images of 3D topological features and thickness histograms of specimens. AFM is also used to determine the number of graphene layers in nano graphitic flakes; however, due to the surface roughness of graphene and variations introduced by AFM, graphene thicknesses are difficult to determine accurately. Kim et al. examined an SLG wrapped Li_4_Ti_5_O_12_ anode in the context of achieving a high Li ion storage capacity [56]. Figure 7a,b show the topological features and a corresponding histogram of graphene oxide [56]. The graphene oxide characterized by AFM was of lateral dimension <5 µm and had a thickness of 1 nm. The thickness indicated by Figure 7b showed that the specimen was monolayer graphene [56].

Prakash et al. studied epitaxial FLG grown at different temperatures using CVD with AFM microscopy [57]. AFM revealed a mesh-like network of atomically flat, tile-like FLG facets. A histogram of FLG thickness revealed thicknesses of 0.7 to 1.2 nm and the average FLG thickness was found to depend on temperature during the growth phase. As temperatures increased from 1350 to 1550 °C, average FLG thickness increased from 0.7 to 3.7 nm [57]. Temiryazev et al. studied graphene and graphite surface contamination at the atomic level using AFM [58] and showed that samples produced under ambient lab conditions had well-ordered layers (lateral dimension > 100 µm^2^ and thickness 4–5 nm) of mostly hydrocarbon species on graphene flake surfaces. Singh et al. also studied the thicknesses of multilayer graphene using AFM [59] and found that multilayer graphene was 1.75 nm thick, which, assuming an interlayer distance of 0.33 nm between adjacent graphene layers, indicated the presence of 6 graphene layers [59].

Yao et al. demonstrated the use of histograms and found that the flake thicknesses of 1, 2, and 4-layer graphene were 1.5, 1.9, and 2.73 nm, respectively, by AFM [60]. Margaryan et al. synthesized large-scale fractal graphene sheets using liquid phase exfoliation [61]. The thicknesses and surface properties of these flakes were determined using AFM imaging, and a flake thickness of 0.35 nm showed that monolayer graphene was produced [61]. Bartlam functionalized graphene and determined the thicknesses of flakes produced by AFM [62]. They found that the thickness of unfunctionalized reduced graphene oxide (U-rGO) was 1.5 nm, that of 4-(4,5a1-dihydropyren-1-yl)butane-1-sulfonate (PBS) functionalized -rGO was 2 nm, and that of sodium 4-(7-cyano-4,5a1-dihydropyren-1-yl)butane-1-sulfonate (PCNBS) functionalized -rGO was 2 nm. Assuming an interlayer spacing of 0.33 nm between adjacent graphene layers, all three produced exfoliated graphene oxide (eGO) flakes were found to be 5 layers thick (U-rGO) and PBS-rGO flakes and PCNBS-rGO were found to be 7 layers thick [62].

### 4.4. Optical Microscopy

**Figure 8:**
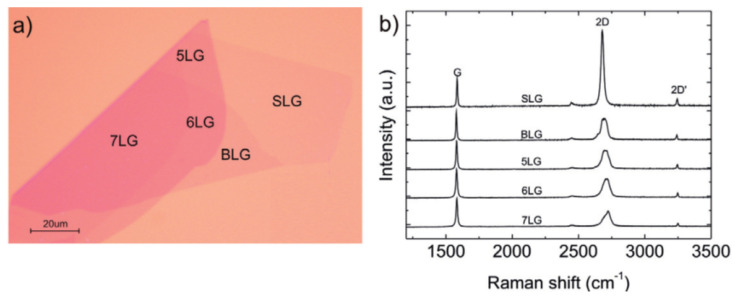
(**a**) Use of optical microscopy to determine numbers of graphene layers in flakes; (**b**) corresponding Raman spectra confirming optical microscopy results (reproduced with permission from ref. [33], Copyright 2012, Elsevier).

Optical microscopy which is also known as light microscopy is often used to study morphologies at low magnifications. This type of microscope uses visible light and a system of lenses to generate magnified images of small samples, but it can also be used to estimate layer numbers per flake. Figure 8a shows single-layer, bilayer and, 5-, 6-, and 7-layer graphenes. Corresponding Raman spectra are shown in Figure 8b [33]. Luo et al. studied single-layer graphene oxide flakes using optical microscopy [63] as an alternative to AFM or SEM, which are considerably more expensive [63]. The used method allows graphene oxide to be imaged over a much larger area (>200 nanosheets per image) than possible with AFM or SEM [63]. The authors concluded that optical imaging could be used as a low-cost, efficient means of determining layer numbers [63]. Campanelli et al. further extended the use of optical microscopy to estimate mono- and bi-layer graphene sheets produced on Q5 and Q20 substrates by micromechanical cleavage [64].

### 4.5. Plasmon Exciton Coupling Spectroscopy

**Figure 9:**
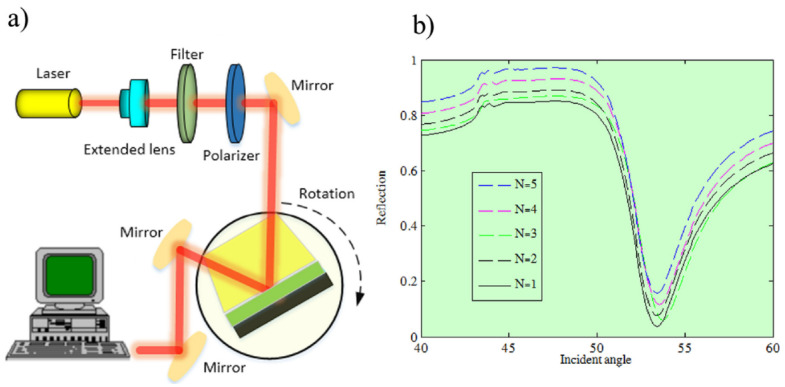
Plasmon exciton coupling spectroscopy technique and the determination of graphene layer numbers: (**a**) experimental set-up, (**b**) experimental results obtained for 1 to 5 graphene layers (reproduced with permission from ref. [65], Copyright 2020, Elsevier).

A plasmon is a quantum of plasma oscillation and can be considered as a quasiparticle arising from the quantization of plasma oscillations. Figure 9a describes an experimental setup used to estimate graphene layer numbers of flakes [65]. In the proposed setup, the Kretschmann configuration (total internal reflection configuration) was used. The setup had three main functional components, namely, a source of incident light, a total internal reflection component, and reflected light detector. Functional details have been previously described [65]. Figure 9b shows the reflection spectra of FLG flakes with different layer numbers [65]. Reflection spectra showed a dip indicating red shift at an angle of incidence of 53°. These experimental results were consistent with the numerical results [65].

### 4.6. X-ray Diffraction

XRD is based on the differential diffractions of an incident X-ray beam caused by sample crystal structures. Using the intensities and angles of diffractions, 3D images of electron densities within crystals are generated. XRD was recently used to determine layer numbers in graphene flakes [66]. Mauro et al. used XRD to study six graphitic materials with different graphene layer numbers [66]. The authors used the full width half maximum (FWHM) of the 002 peak in Scherrer’s equation to determine graphene flake thicknesses [66], which were then divided by interlayer distances (0.34 nm) to obtain layer numbers of around 60, 40, and 30 for samples A, B, and C. Galimberti et al. studied XRD for determining the number of graphene layers stacked in nanographite flakes [67]. The authors demonstrate that using FWHM of the (002) peak, which is the characteristic peak for carbon-based materials, the number of layers can be determined. The use of Scherrer’s equation to determine nanographite flake thickness found it to be 9.8 nm. Considering an interlayer spacing of 0.339 nm, a total of 29 layers were estimated [67]. Galimberti et al. gained further insight through the use of XRD to estimate shape anisotropy. For that, the lateral dimension of nanographite flake FWHM was determined though (100) and (110) reflections and a lateral length of 30.2 nm was estimated. The shape anisotropy was then calculated by dividing the lateral length with flake thickness and a value of 3.1 was determined [67]. Kumar et al. studied the used XRD to determine the number of graphene layers stacked in graphene flakes and total numbers of 45–48 layers were estimated [68].

### 4.7. Raman Spectroscopy

**Figure 10:**
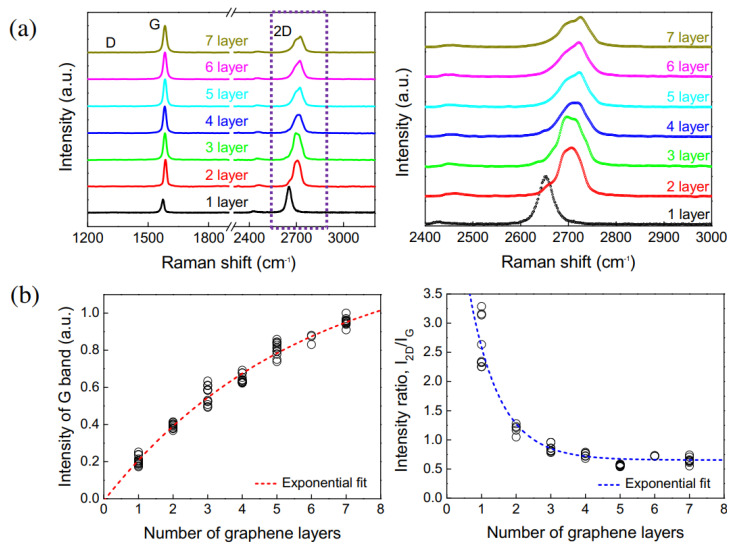
Raman spectra of 1 to 7 layered graphenes: (**a**) spectral evolution; (**b**) Intensities of G-bands and Intensity ratios of 2D vs. G bands (reproduced with permission from ref. [40], Copyright 2014, Elsevier).

Raman spectroscopy provides information on the vibrational modes of molecules, and is frequently used to obtain the structural fingerprints of molecules, but it has also been used to determine graphene layer numbers in flakes. The most prominent spectral feature of carbon-based materials is the appearance of a D-band, G-band and a 2D band [69] (Figure 10a). Furthermore, the shape of the 2D band changes as the graphene layers increase from a monolayer up to 7 layers [40]. Moreover, the intensity ratio of 2D vs. G bands decreases from 3.3 for single layer graphene to nearly 0.5 for 7-layer graphene [40]. The number of graphene layers can be easily determined from the position and shape of the 2D band. For example, the 2D-band peak position shifted to higher numbers, i.e., from 2702 cm^−1^ for 3-layer graphene to 2720 cm^−1^ for 10-layer graphene and 2725 cm^−1^ for a graphite of thickness 40 nm [41]. Figure 10b shows that the I_D_ and I_2D/G_ band depend on layer number. For example, the intensity of the D-band increases with layer number, and the intensity ratio of the 2D/G–band decreases with layer number [40]. Güler et al. studied the exfoliation process of graphite under mild sonication in different solvents [70] and optimized the sonication time and power to produce highly exfoliated graphene sheets. The extent of exfoliation was determined by Raman spectroscopy [70]. The Raman spectra confirmed the formation of graphene based on the shape, location, and intensity of the 2D peak, which are dependent on layer number.

Niavol et al. studied a single layer graphene-based sensor for monitoring toxic gases [71] and confirmed the presence of single-layer graphene, before and after deposition of a NiO layer, using Raman spectroscopy based on 2D-band peak intensity [71]. Schiliro et al. studied the nucleation of aluminum oxide deposited on epitaxial graphene on silicon carbide, and determined the layer numbers using various techniques including Raman spectroscopy [72]. Unlike other types of graphene, nucleation sites of uniform density were observed for silicon carbide with 98% coverage of monolayer graphene and 2% coverage with bi-tri layer graphene. Gong et al. studied the friction of FLG (mono-, bi-, and tri-layer) on silica and silica oxide substrates using Raman spectroscopy [73].

Haehnlein et al. investigated epitaxial graphene of different types, such as defective/single-layer graphene to multilayer graphene with small defect densities by Raman spectroscopy and determined graphene layer numbers using strain types [74]. A novel correlation was observed between the 2D mode line width and the inverse I_D_/I_G_ ratio, which allowed the determination of strain type and layer number [74]. Kolomiytsev et al. employ Raman spectroscopy to investigate the layer numbers of multilayer graphene, and found that there were 10 layers per multilayer graphene flake [75]. Yitzhak et al. studied fluctuations in Raman spectra for FLG and single-layer graphene and analyzed the positions and intensities of the Raman peaks for different layer numbers [76]. The average positions of Raman spectra lines were shifted in opposite directions, which made it possible to differentiate bilayer and monolayer films, despite similar Raman spectra [76].

Silva et al. used Raman spectra to analyze graphene layer numbers of mass-produced flakes using 2D- and G-bands. The 2D-band provided information about exfoliation in graphene flake whereas the intensity of the G band provided information about layer numbers [77]. Li et al. prepared high concentrations of two types of graphene slurries and studied their rheological studies, e.g., viscosity, graphene loading, surfactant content and different solvents, and temporal effects. Raman spectroscopy was also used to determine layer numbers of graphene flakes and showed graphene grade 1 had ≥1 to ≤5 layers and that grade 2 graphene had a 5-layer graphitic structure [78].

Papanai et al. examined graphene layer numbers of mechanically exfoliated graphene flakes using Raman spectroscopy [79]. The authors used intensity ratios (I_2D_/I_G_) and peak widths of the 2D-band to determine layer numbers. The 2D peak of single-layer graphene (SLG) consisted of a single Lorentzian peak with a width of 24 cm^−1^, but this was split into four, six, and five components for 2-, 3-, and 5-layer graphenes, respectively [79]. Niilisk et al. used Raman spectroscopy to investigate the multilayer stacking of graphene grown on Ni [80]. Raman spectra showed several combinations of in-plane and out-of-plane Raman modes, indicating the formation of many multilayer graphene domains where graphene layers were found stacked or in an exfoliated form in the flake [80]. Maruyama et al. demonstrated liquid phase growth of FLG on sapphire at different temperatures using SiC micro powder as a source material. Raman spectroscopy was used to determine the qualities, defect, and numbers of stacked graphene layers [81]. Results showed layer numbers ranging from 1 to 3 and it was confirmed that the synthesized flakes were of FLG [81].

### 4.8. Raman Mapping

**Figure 11:**
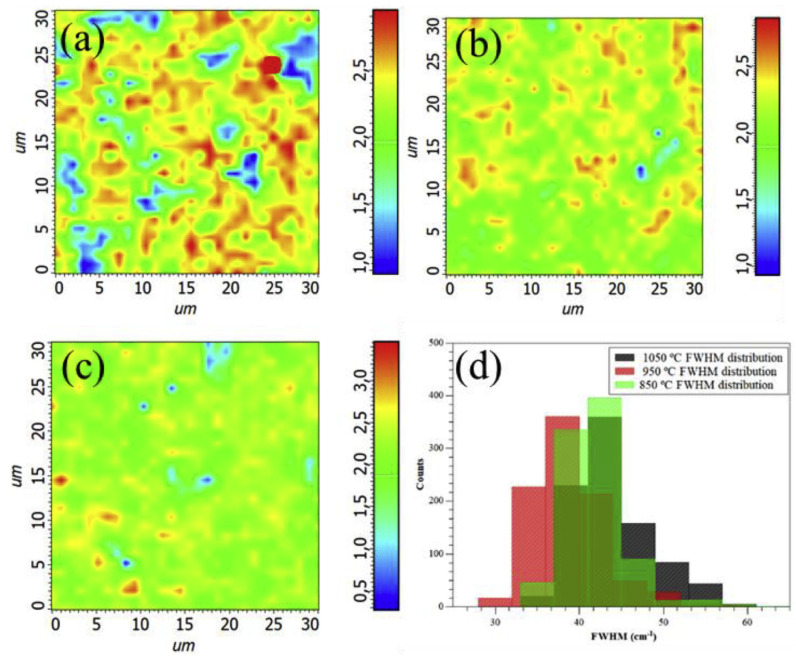
Raman maps used for determining graphene layer numbers in flake samples prepared at different temperatures (**a**) 850 °C, (**b**) 950 °C, or (**c**) 1050 °C. (**d**) Histograms of 2D-band of samples prepared at different temperatures (reproduced with permission from ref. [82], Copyright 2018, Elsevier).

Raman mapping is a laser-based microscopic technique used to obtain performing Raman spectra. Recently, Raman mapping was used to determine graphene layer numbers and homogeneities of graphene flakes. Barbosa et al. prepared single-layer graphene using oxygen-free N-octane as a precursor [82]. Graphene heterogeneity was higher at 850 °C than at 950 °C (Figure 11a,b, respectively), which revealed the presence of 2-layer graphene or FLG containing a predominance of single-layer graphene. However, the Raman map in Figure 11c obtained at 1050 °C, showed that the sample was the most homogenous [82]. Figure 11d shows the FWHM values of the 2D-bands of different graphene samples prepared at different temperatures. Kim et al. highlighted the direct formation of graphene by ion implementation on Cu, Ni and Cu/Ni alloy [83] and achieved better graphene coverage and quality for the alloy, which was attributed to the greater carbon diffusivity and lower carbon solubility of the alloy than for Ni, and a lower activation energy than Cu [83].

Raman mapping was used to determine the thicknesses and graphene layer numbers of synthesized flakes. Raman maps of CuNi alloy after annealing at 900 °C for 30 min showed clear G and 2D modes. Coverage was approximately 63% of 10-10 µ^2^ area [83]. Bayram studied 3D graphene films synthesized on glass/FTO by Raman mapping and showed Raman mapping of D, G and 2D peaks showed that there exist homogeneously on all surfaces. In addition, mapping also revealed homogenous morphology and significant graphene film peaks [84]. Shen et al. studied the synthesis of bilayer graphene on Cu substrate using a hot filament CVD technique [85] and reported the synthesis of homogenous bilayer graphene of superior lubricity and higher wear resistance of potential use for various industrial applications [85]. The Raman mapping was involved in the I_D_/I_G_ and the I_2D_/I_G_ ratio to study the homogeneity of the synthesized bilayer graphene and demonstrated the high uniformity of bilayer graphene over deposited areas [85]. Wang et al. studied graphene properties using Raman maps under different strains to investigate their suitability for a wide range of applications, such as in flexible electronics [86]. Raman maps showed that strains of 0.39% and 0.8% produced no evidence of wrinkling or inhomogeneity inside domains when applied in different directions. It further highlights the strain uniformity >85% for domains further demonstrate a good adhesion between graphene and the substrates [86]. The studies further provided insight that certain strains show no domain boundary effect or strain localization was observed [86]. 

Woo et al. prepared graphene using CVD and used Raman maps on channels of graphene to study the uniformities in the graphene layers. Raman maps of individual graphene flakes showed that, in addition to the FWHM of 2D peak, the G peak position showed a lighter color at the edges compared with inside the graphene channels [87]. Liu et al. studied the thermal stability of graphene at temperatures of 600, 800, and 1000 °C in an inert atmosphere. Raman maps showed a continuous and uniform distribution of graphene on Si wafers and different brightnesses of graphene layers; bright areas had a graphite-like structures while dark areas corresponded to single-layer graphene. Furthermore, they reported that most areas in Raman maps corresponded to single-layer graphene, though a few defects in single-layer graphene were also observed [88]. Amato et al. grew mechanically stable graphene using CVD on Co and successfully transferred graphene from the substrate without using a polymeric support. However, strain in graphene sheets was observed after transfer. These sheets were analyzed for homogeneity by Raman mapping [89], and the obtained results showed that 50% of the areas investigated were covered with bi-layer graphene. The origin of strain in synthesized graphene was further investigated by Raman mapping on Co. The presence of two holes was observed in the Co film beneath the graphene layer. Oh et al. studied single and bilayer graphene and their uniformities using Raman maps of 2D peaks and observed defects of transferred mono-bi-graphene sheets, such as holes and wrinkles [90].

### 4.9. Spin Hall Effect

**Figure 12:**
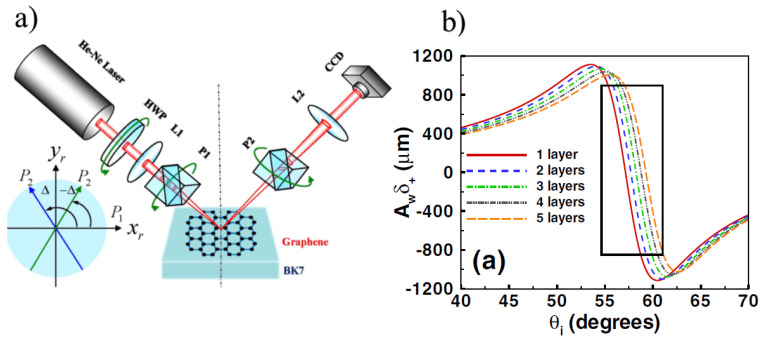
(**a**) Experimental setup, BK7 is glass with a transferred graphene film, L1 and L2 are lenses, HWP is a half-wave plate used to adjust intensity, P1 and P2 are Glan laser polarizers, CCD is a charge-coupled device; (**b**) determination of graphene layer numbers using the Spin Hall Effect (reproduced with permission from ref. [91], Copyright 2012, AIP).

The spin Hall Effect is a transport phenomenon, which manifests as spin accumulation on the lateral surfaces of electric-current-carrying specimens and opposing surface boundaries with spins of opposite signs. Figure 12a shows an experimental setup used to determine the number of graphene layers in flakes using the spin Hall Effect. A theoretical relation has been established between transverse shift and layer numbers and confirmed experimentally [91]. Figure 12b shows that layer numbers can be determined using the spin Hall Effect. Transverse displacements were studied at different angles of incidence and were found to be related to layer numbers from 1 to 5 [91]. Layer numbers determined using this method were confirmed by Raman spectra [91].

### 4.10. Scanning Tunnelling Microscopy

**Figure 13:**
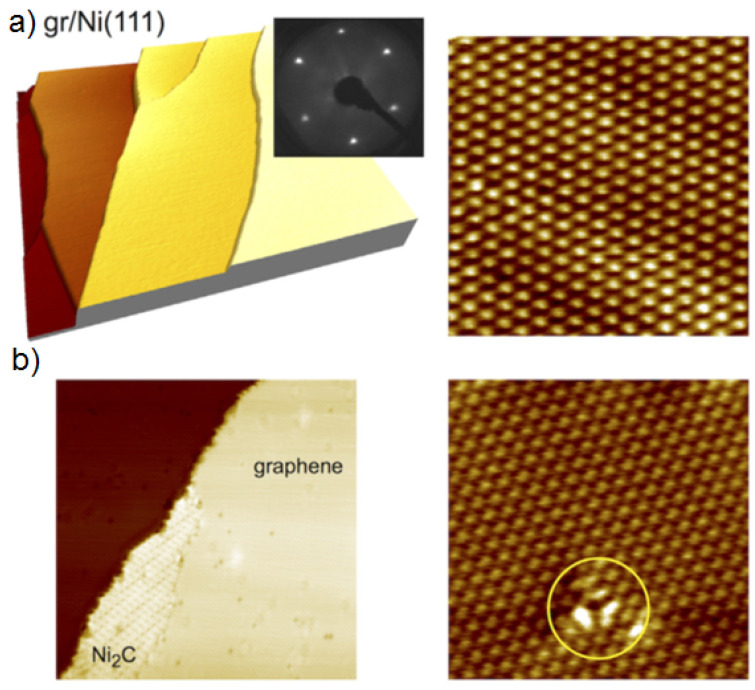
(**a**) Use of STM for the determination of graphene layer numbers per flake, (**b**) STM image showing defects in a graphene flake (reproduced with permission from ref. [92], Copyright 2017, Elsevier).

STM provides high-resolution images of surfaces at the atomic level. The technique involves sensing the surface of a specimen with a sharp conducting tip. Resulting images can capture features smaller than 0.1 nm and have a depth resolution of 0.01 nm. Figure 13a shows a STM image of a well-ordered graphene layer with a small carbide phase [92]. The image reveals a defect-free, well-defined structure. The high quality of graphene/Ni(111) was confirmed using LEED images (inset of Figure 13a). Figure 13b shows a STM image of graphene defects (missing carbon atoms), which are presumed to have been caused by oxygen intercalation [92].

### 4.11. Scanning Electrochemical Microscopy

SCEM is used to measure local electrochemical behavior at different interfaces. Interpretation of SCEM technique is based on the concept of a diffusion-limited current. Wain et al. presented topological features, as observed by AFM-SECM [93]. In graphene regions, Wain et al. showed examples of FL, ML, and SL. The authors also demonstrated the use of a SECM-AFM cantilever probe for high-resolution topological-electrochemical probe microscopy. The probing provided excellent topological-electrochemical mapping and enables the determination of graphene layer numbers per flake, such as for FL, ML and SL. This study, demonstrated that the AFM-SECM technique provides insights of the interfacial behavior of exfoliated graphene flakes [93]. The summary of the type of graphene, method of synthesis, method of investigation and references are described below in Table 1.

## 5. Conclusions

Graphene flakes have remarkable effects on the physicochemical properties of graphene-based nanocomposites. Graphene layer numbers can be characterized microscopically, spectroscopically, or using plasmon exciton coupling spectroscopy, or the spin hall effect. Graphene can be synthesized using several bottom-up or top-down approaches, and because of its superior properties it has many applications, especially in the flexible electronics field. The stacking of monolayer graphene to form few-layer or multilayer graphenes significantly influences the properties of graphene. This review summarizes various techniques used to determine the layer numbers of graphene flakes. Greater layer numbers from monolayer graphene to few-layer graphene and multilayer graphene were found to significantly diminish the properties of graphene. This review summarizes the use of graphene with different layer numbers for various applications (flexible electronics, tissue engineering, biosensors, energy harvesting, actuation and strain sensors) and provides guidance regarding the effects of graphene flake thickness on the physicochemical properties of graphene.

## Figures and Tables

**Table 1:** Summary of type of graphene, method of synthesis, method of investigation and the reference.

S. No.	Type of Graphene	Method of Synthesis	Method of Investigation	Reference
1.	Monolayer to few-layer to multilayer graphene	-	Optical microscopy, Raman spectra	[37]
2.	Monolayer to few-layer to multilayer graphene	-	Optical microscopy, Raman spectra	[40]
3.	Graphite-like thin sheets	-	TEM	[45]
4.	1–5-layer thick graphene layers	Ultrasonication-induced exfoliation of graphite to graphene in solvent	TEM	[46]
5.	Reduced graphene oxide	Chemical synthesis	XRD, TEM, and other electron spectroscopic methods	[47]
6.	Monolayer graphene	CVD	TEM	[48]
7.	Mono-bilayer graphene	High sulfonic acid edge functionalized graphene from graphite	HR-TEM	[49]
8.	Mono-few-layer graphene	CVD	SEM, Optical, Raman spectra, Raman mapping	[50]
9.	Single or multilayer Graphene flakes	CVD	SEM, Optical, Raman spectra, Raman mapping, and AFM	[51]
10.	Graphene layers stacked 6 to 20 layers thick	Ball milling	SEM, Raman spectra, HRTEM	[52]
11.	Monolayer graphene	CVD	SEM, Optical, microscopy, Raman spectra, Raman mapping, and AFM	[53]
12.	Few-layer graphene	-	SEM, Optical microscopy, HRTEM, Raman mapping, and AFM	[54]
13.	Monolayer graphene	CVD	SEM, Raman spectra, Raman mapping, and AFM.	[55]
14.	Monolayer graphene	Chemical synthesis	AFM	[56]
15.	Few-layer graphene	CVD	AFM	[57]
16.	Graphene flake with thickness in 4–5 nm	-	AFM	[58]
17.	Multilayer graphene	-	AFM	[59]
18.	Monolayer to few-layer graphene	-	AFM	[60]
19.	Monolayer graphene sheet	Liquid phase exfoliation	AFM	[61]
20.	Functionalized reduced graphene	Chemical synthesis	AFM	[62]
21.	Monolayer graphene oxide	Chemical synthesis	Optical microscopy, AFM, SEM	[63]
22.	Mono-bilayer graphene	Chemical synthesis	Optical microcopy	[64]
23.	Mono-few-layer graphene	-	Plasmon exciton coupling spectroscopy	[65]
24.	Graphene flake with 60, 40 and 30 layers thick	-	X-ray diffraction	[66]
25.	Exfoliated graphene sheets	Graphite sonication in solvents	Raman spectra	[70]
26.	Monolayer graphene	CVD	Raman spectra	[71]
27.	Epitaxial graphene	CVD	Raman spectra	[72]
28.	Few-layer graphene	CVD	Raman spectra	[73]
29.	Monolayer to multi-layer graphene	-	Raman spectra	[74]
30.	Multilayer graphene	-	Raman spectra	[75]
31.	Monolayer to few-layer graphene	-	Raman spectra	[76]
32.	Exfoliation in graphene flake	-	Raman spectra	[77]
33.	Monolayer to few-layer graphene		Raman spectra	[78]
34.	Monolayer to few-layer graphene		Raman spectra	[79]
35.	Multilayer graphene	CVD	Raman spectra	[80]
36.	Mono to few-layer graphene	Liquid phase exfoliation	Raman spectra	[81]
37.	Monolayer graphene	CVD	Raman mapping	[82]
38.	Graphene flake	CVD	Raman mapping	[83]
39.	3D graphene films	CVD	Raman mapping	[84]
40.	Bilayer graphene	CVD	Raman mapping	[85]
41.	Graphene flake	-	Raman mapping	[86]
42.	Graphene flake	CVD	Raman mapping	[87]
43.	Monolayer graphene	-	Raman mapping	[88]
44.	Mono-bilayer graphene	CVD	Raman mapping	[89]
45.	Mono-bilayer graphene	-	Raman mapping	[90]
46.	Mono-few-layer graphene	-	Spin hall effect, Raman spectra	[91]
47.	Mono-bilayer graphene	-	Scanning tunneling microscopy	[92]
48.	Mono-few–multilayer graphene	-	Scanning electrochemical microscopy and AFM	[93]

## Data Availability

No new data were created or analyzed in this study, data sharing is not applicable to this article.
